# Identification of cuproptosis-related lncRNA prognostic signature for osteosarcoma

**DOI:** 10.3389/fendo.2022.987942

**Published:** 2022-10-13

**Authors:** Binfeng Liu, Zhongyue Liu, Chengyao Feng, Chenbei Li, Haixia Zhang, Zhihong Li, Chao Tu, Shasha He

**Affiliations:** ^1^ Department of Orthopaedics, The Second Xiangya Hospital, Central South University, Changsha, China; ^2^ Hunan Key Laboratory of Tumor Models and Individualized Medicine, The Second Xiangya Hospital of Central South University, Changsha, China; ^3^ Department of Oncology, The Second Xiangya Hospital, Central South University, Changsha, China

**Keywords:** osteosarcoma, cuproptosis, metabolism, lncRNA, immunotherapy, tumor microenvironment

## Abstract

**Background:**

Copper is an indispensably mineral element involved in various metabolic processes and functions in the active sites of many metalloproteins. Copper dysregulation is associated with cancers such as osteosarcoma (OS), the most common primary bone malignancy with invasiveness and metastasis. However, the causality between cuproptosis and OS remains elusive. We aim to identify cuproptosis-related long non-coding RNAs (lncRNAs) for osteosarcomatous prognosis, immune microenvironment response, and immunotherapy.

**Methods:**

The Person correlation and differential expression analysis were used to identify differentially expressed cuproptosis-related lncRNAs (CRLs). The univariate, least absolute shrinkage and selection operator (LASSO), and multivariate Cox regression analysis were performed to construct the CRL signature. The Kaplan–Meier (K-M) survival analysis, receiver operating characteristic (ROC) curve, internal validation, independent prognostic analysis, and nomograph were used to evaluate the prognostic value. The functional enrichment, tumor microenvironment, immunotherapy and chemotherapy response between the two distinct groups were further explored using a series of algorithms. The expression of signature CRLs was verified by real-time quantitative polymerase chain reaction (RT-qPCR) in OS cell lines.

**Results:**

A novel CRL signature consisting of four CRLs were successfully identified. The K-M survival analysis indicated that the OS patients in the low-risk groups had a better prognosis than that in the high-risk group. Then, the ROC curve and subgroup survival analysis confirmed the prognostic evaluation performance of the signature. Equally, the independent prognostic analysis demonstrated that the CRL signature was an independently predicted factor for OS. Friends analysis determined the hub genes that played a critical role in differentially expressed genes between two distinct risk groups. In addition, the risk score was related to immunity status, immunotherapy response, and chemotherapeutic drug sensitivity. Finally, the expression of these signature CRLs detected by RT-qPCR was consistent with the bioinformatic analysis results.

**Conclusion:**

In summary, our study confirmed that the novel CRL signature could effectively evaluate prognosis, tumor immune microenvironment, and immunotherapy response in OS. It may benefit for clinical decision-making and provide new insights for personalized therapeutics.

## Introduction

Osteosarcoma (OS) is a malignant tumor derived from mesenchymal cells, which most commonly occurs in children and adolescents (1). As there is no apparent clinical manifestation in early OS, the most diagnosed patients are already in advanced stages, accompanied by distant metastasis (2). Currently, the long-term survival rate for the localized OS is approximately 68%, while it is still less than 30% in patients with recurrence or metastasis (3, 4). Meanwhile, the treatment for OS has been limited improved over the last three decades, especially in patients with multidrug resistance, recurrence or lung metastasis (5, 6). This is mainly attributed to the lack of knowledge of the pathogenic mechanisms (7). To this end, it is urgent to disclose effective prognostic biomarkers and promising signatures.

Long non-coding RNA (lncRNA) refers to non-coding RNAs longer than 200 nucleotides, a large class of gene transcripts encoded by the genome, but most do not encode proteins (8). Accumulating evidence shows that lncRNAs play critical roles in regulating cancer proliferation, metastasis, cycling, and programmed death (9–11). LncRNA HIF2PUT was demonstrated to inhibit the proliferation, migration, and invasion of osteosarcoma cells by regulating HIF2 expression (12). Iron metabolism-related lncRNA signature has also been proved to predict survival outcomes for OS (13). For instance, Zhijie Xu et al. demonstrated that the ferroptosis-related lncRNA signature could precisely predict prognosis and immune response for hepatocellular cancer (14). Therefore, metal ions regulated lncRNA signature would be prognostic candidates for OS.

Copper is an intracellular trace metal that plays an integral role in various metabolic processes. Cuprotosis is a new form of precisely regulated programmed cell death, wherein excess intracellular copper induces proteotoxicity and dysfunction of the mitochondrial tricarboxylic acid (TCA) cycle *via* lipoylated dihydrolipoamide S-acetyltransferase (DLAT) aggregation (15) LncRNAs were implied as a crucial role in copper-induced toxicity (16). Therefore, we reasoned that cuproptosis-related lncRNAs (CRLs) might be a promising biomarker for OS. Here we report the identification of prognostic CRL signature, the underlying mechanism, and *in vitro* experimental validation. This could be used for individualized survival prediction and to develop more effective strategies for systemic treatment.

## Materials and methods

### Data sources

To explore differentially expressed CRLs in OS, we downloaded the GSE126209 dataset from the Gene Expression Omnibus (GEO, https://www.ncbi.nlm.nih.gov/geo/) database. The mRNA and lncRNA data of six normal and OS tissues were extracted from GSE126209 for further study. Meanwhile, the gene profile and corresponding clinical information of OS patients were downloaded from the Therapeutically Applicable Research To Generate Effective Treatments (TARGET, https://ocg.cancer.gov/programs/target) database for the subsequent CRLs signature construction and related bioinformatics analysis. These expression data were further normalized by log2 (expression + 1) transformation. The detailed clinical information of the OS cohort from the TARGET database is shown in **Table S1**. The above expression profile was normalized to remove nonbiological impact and correct for systematic data. The cuproptosis-related genes in this study were extracted from a previous paper (15) and contained ten cuproptosis-related genes. The detailed information of these genes is displayed in **Table S2**.

### Differential expressed lncRNA in OS

The principal component analysis (PCA) was utilized for OS tissue and normal tissue to visualize the differences in expression patterns. To identify differentially expressed lncRNAs between OS and normal tissue, we performed the differential analysis utilizing package “limma” in R software (17). The screening thresholds were |log2FC| ≥ 1 and adjusted P-value < 0.05.

### Identification of CRLs in OS

We conducted the Pearson co-expression analysis to obtain differentially expressed CRLs in OS. Initially, Spearman correlation coefficients were calculated based on the expression value of cuproptosis-related genes and each lncRNA to identify CRLs (|R^2^|>0.3 and p<0.05) (18). Subsequently, the CRLs were intersected with those mentioned above differentially expressed lncRNAs to select differentially expressed CRLs for further investigation.

### Screen CRLs are associated with the prognosis of OS

After selecting the differential expressed CRLs, the univariate COX analysis was performed to identify the CRLs associated with the prognosis of OS. The prognostic CRLs with p-value < 0.05 were screened as candidate lncRNAs for the cuproptosis-related lncRNAs prognostic signature and following analysis.

### Construction and validation of the CRL signature

The OS cohort was randomly separated into a training cohort and a testing cohort in a 1:1 ratio using the “caret” package. The training cohort was used for signature building, while the testing cohort and the entire cohort were employed for validation. Primarily, the prognosis-related CRLs are subject to the least absolute shrinkage and selection operator (LASSO) Cox regression analysis to narrow down the candidate CRLs. Subsequently, the Multivariate Cox regression analysis was performed to further screen genes to optimize the CRL prognostic signature. The cuproptosis-related prognostic risk scores of each OS patient were calculated using the following formula: risk score = Σ (Coef_i_ * Exp_i_), where Coef_i_ and Exp_i_ represent the corresponding coefficient and expression level of each lncRNA, respectively. Next, the OS patients in the training cohort were assigned to low-risk and high-risk groups according to the median risk score in the training cohort. The Kaplan–Meier (K-M) survival analysis compared the overall survival between the two distinct risk groups. The receiver operating characteristic (ROC) curve was further drawn to assess the predictive accuracy of the CRL prognostic signature. The risk score of each OS patient in the testing cohort and the entire cohort was calculated according to the same formula. Then, the same methodology described above was conducted to evaluate the potential and applicability of the novel signature. For each CRLs included in the novel prognostic signature, their expression levels, their relationship with cuproptosis-related genes, and their respective correlations were further explored.

### Prognostic and independent analysis

The risk scores are combined with clinical information for subsequent investigation, including survival time, survival status, gender, age, metastatic status, and tumor location. To exclude the possible confounding effects from other clinical factors, we performed a subgroup survival analysis according to the age, gender, and metastasis status of the OS. Subsequently, the univariate and multivariate Cox analyses were used to determine whether the novel CRLs signature had an independent prognostic ability for OS.

### Differential expression analyses between distinct risk groups

To assess differences in expression profiles between high-risk and low-risk subgroups, the differentially expressed genes between the two distinct risk groups were identified by using the limma package (|log2FC|>0.585 and FDR<0.05). The volcano plot and clustered heat plot were used further to display the result of the differentially expressed analysis. In addition, the distribution of patients with different risk scores was displayed by utilizing PCA.

### Function enrichment analysis

To further explore the functional differences between the differential genes in the distinct risk groups, we used the “clusterProfiler” package to carry out Gene Ontology (GO) and the Kyoto Encyclopedia of Genes and Genomes (KEGG) enrichment analysis (19). The GO analysis included the Biological Process (BP), Cellular Component (CC), and Molecular Function (MF). Further, the bubble diagrams were used to visualize the enrichment results. The adjusted nominal P-values < 0.05 was selected as thresholds of significance.

### Protein-protein interaction network and friends analysis

Next, a PPI network was constructed to identify hub genes among the differentially expressed genes using the String database (https://cn.string-db.org/cgi/input) with default parameters. Meanwhile, the PPI network was visualized using Cytoscape software (version 3.9.0). In addition, the Friends analysis was further performed using the “GOSemSim” package to screen out hub genes (20). Ultimately, the top ten genes screened by Friends analysis were visualized and presented as hub genes for subsequent analysis.

### The expression and prognosis value of top ten hub genes

After obtaining the cuproptosis-related hub genes based on the above analysis, we further analyzed their function in OS. The co-expression analysis was used to explore the association between hub genes and cuproptosis. Additionally, the K-M survival and ROC curves were further used to evaluate the prognostic performance of the top 10 hub genes in OS.

### Gene set enrichment analysis and gene set variation analysis

To further explore the molecular and biological differences between high and low-risk groups, we performed GSEA analysis by package “clusterProfiler”. The KEGG dataset was extracted from the molecular signature database (https://www.gsea-msigdb.org/gsea/msigdb), and the p < 0.05 were selected as thresholds of significance. The GSVA was further performed to analyze the enrichment of biological processes and pathways due to CRLs risk level through package “GSVA “ in R (21). Subsequently, the “limma” package was used to perform the differential analysis to screen the significantly different pathways(|log2FC|>0.1 and P-value<0.05). The screened significantly different pathways were visualized as a clustered heatmap.

### Assessment of immune microenvironment and immune cell infiltration

Currently, several reliable approaches have been established for quantifying immune infiltration and the immune microenvironment in tumors based on transcriptome data, such as Estimation of Stromal and Immune Cells in Malignant Tumors using the Expression Data (ESTIMATE) (22) and single sample gene set enrichment analysis (ssGSEA) algorithm (23). In our study, we calculated and compared the immune microenvironment and immune-infiltrating cell content between high-risk and low-risk groups. The ESTIMATE is an algorithm that can detect the tumor purity and activity of immune and stromal cells in the immune microenvironment by transforming gene expression. Briefly, the ESTIMATE algorithm was used to calculate the immune score of each OS patient and then compare the difference in ESTIMATE score between the high- and low-risk groups. Also, infiltration levels of 28 immune cells were inferred by the ssGSEA algorithm and compared in the different risk groups.

### Immunotherapy response and drug sensitivity analysis

The immunotherapy response difference was compared using subclass mapping (submap), with lower P-values indicating a higher similarity. Additionally, the “pRRophetic” package was utilized to predict the half maximal inhibitory concentration (IC50) of the chemotherapeutic drug, which indicates the effectiveness of a substance in inhibiting a specific biological or biochemical process (24).

### Nomogram and calibration

To provide a scoring system that predicts the individual probability of patients’ prognosis, we established a prognostic nomogram, which can assess the probable 1‐year, 3‐year and 5‐year survival of OS. Meanwhile, we also drew a calibration curve to validate the predictive value of the constructed nomogram, which visualizes the consistency between the actual result and the probability predicted by the nomogram. The “rms” package was used to plot the nomogram and calibration curve. In addition, the ROC curve was applied to investigate the prognostic value of the nomogram.

### Cell culture

The normal osteoblast cell line (hFOB1.19) was purchased from American Type Culture Collection (ATCC) and cultured in Dulbecco’s Modified Eagle Medium/Nutrient Mixture F12 (DMEM/F12) medium (Gibco, United States). Human osteosarcoma cell lines 143B, HOS, and MG-63 were purchased from ATCC and cultured in a minimum essential medium (MEM) (Gibco, United States). Human osteosarcoma cell line ZOS was gifted by Prof. Kang Tiebang (Sun Yat-Sen University, China) and cultured in Dulbecco’s Modified Eagle’s medium (DMEM) (Gibco, United States). Human osteosarcoma cell line SJSA-1 was purchased from ATCC and cultured in RPMI-1640 medium (Gibco, United States). All the cell lines were cultured with 10% fetal bovine serum (Gibco, United States) and 1% penicillin-streptomycin solution (NCM Biotech, China) besides SAOS-2, which was cultured with 15% fetal bovine serum (Gibco, United States) and 1% penicillin-streptomycin solution (NCM Biotech, China). All the cell lines were cultured in a humidified atmosphere with 5% CO2 at 37°C.

### Real-time quantitative polymerase chain reaction

Total cellular RNA was drawn from cell lines utilizing RNA Express Total RNA Kit (M050, NCM Biotech, China). Next, total RNAs were used for cDNA synthesis with Revert Aid First Strand cDNA Synthesis Kit (K1622, Thermo Scientific, United States). Subsequently, the expression level of each gene was quantified by Hieff qPCR SYBR Green Master Mix (High Rox Plus) (11203ES, YEASEN Biotech Co., Ltd, China) and calculated with the 2-^ΔΔCT^ method. GAPDH was used as the internal reference for normalization. The primer sequences are listed in **Table S3**.

### Statistical analysis

All statistical analyses and plots in the present study were performed in R version 4.0.5. The differences in the expression of signature CRLs between OS and normal cells were assessed using independent t-tests. Spearman correlation analysis was used to determine correlation. The Chi-square test was used to analyze the differences in clinical characteristics between the distinct risk groups. P < 0.05 was defined as statistical significance.

## Results

### Identification of cuproptosis-related differentially expressed lncRNAs

**Figure 1:**
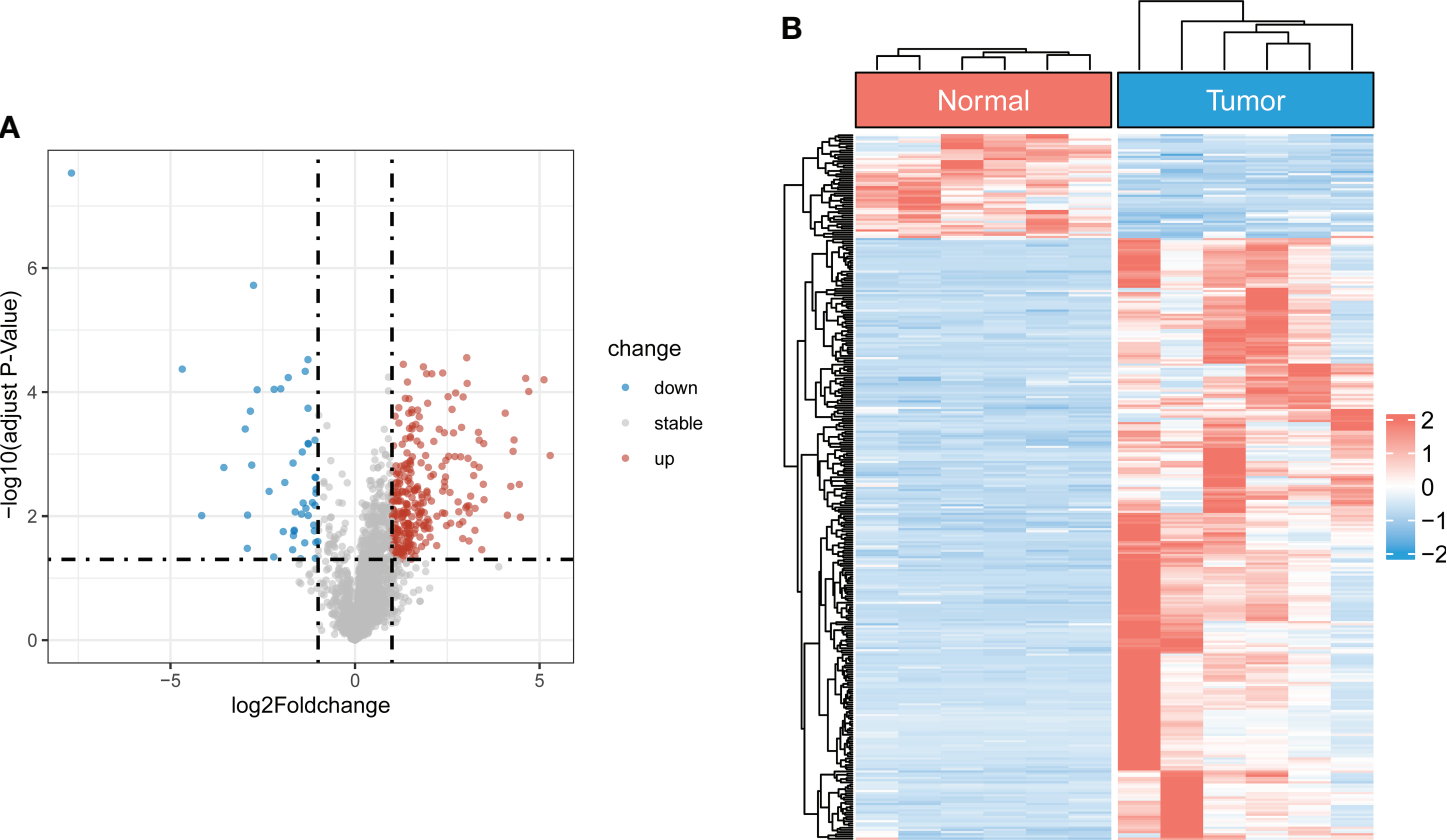
Identification of differentially expressed CRLs in OS **(A)** The volcano plot of differentially expressed lncRNAs. Blue represent down-regulated CRLs, and red represents upregulated CRLs. **(B)** Hierarchically clustered heat maps of differentially expressed lncRNAs. Red and blue represent normal and tumor tissues, respectively.

The flowchart of the study is illustrated in **Figure S1**. After batch effect correction and normalization, the mRNA and lncRNA expression profiles were extracted (**Figure S2**). Based on the enrolled criteria, a total of 326 differentially expressed lncRNAs between the OS tissue and normal tissue were identified, of which 278 were upregulated, and 48 were downregulated (**Figures 1A, B**). Then, Spearman correlation analysis was conducted between lncRNAs and cuproptosis-related genes in the OS According to the inclusion parameters of correlation coefficient (|R^2^|) > 0.3 and p-value <0.05, there were 307 CRLs identified in OS (**Table S4**). Taken together, all CRLs were differentially expressed in OS (**Table S5**).

### Derivation and validation of a CRLs prognostic signature

**Figure 2:**
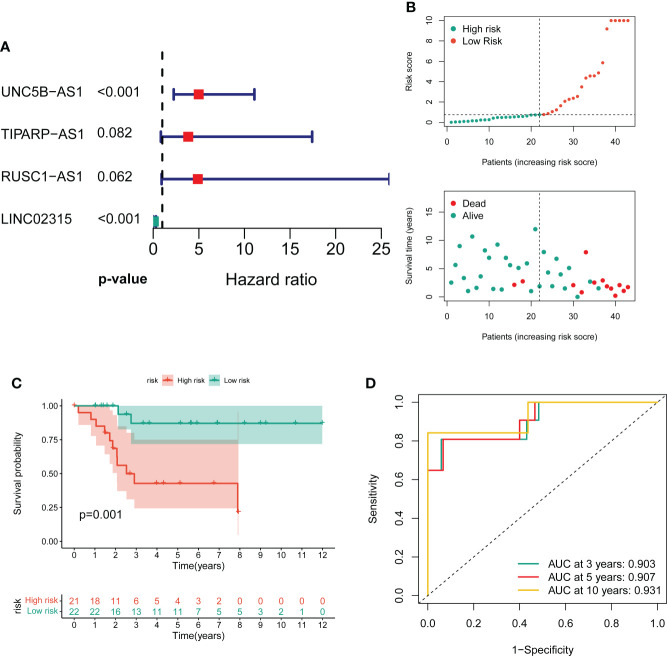
Construction of the novel CRL signature. **(A)** Forest plot of multivariate cox regression analysis for prognostic genes. **(B)** The distribution of the risk scores and the distributions of overall survival status and risk score in the training groups. **(C)** Kaplan–Meier analysis of the overall survival between the two distinct risk groups. **(D)** ROC curves to predict the sensitivity and specificity of 3-, 5-, and 10-year survival according to the novel cuproptosis-related signature.

To screen the prognostic value of differentially expressed CRLs, we performed the univariate Cox regression analysis to explore the association between the CRLs and the overall survival of OS. As a result, 31 prognostic CRLs were identified for signature construction (**Table S6**). Then, these prognostic CRLs were subject to the LASSO method in the training group to determine candidate signature CRLs (**Figure S3**). Next, the multivariate Cox regression analysis identified the optimal CRLs prognostic risk signature composed of UNC5B-AS1, TIPARP-AS1, RUSC1-AS1 and LINC02315 (**Figure 2A**). In the novel CRLs signature, the corresponding cuproptosis risk score of each OS patient was calculated as the following formula: Risk Score = UNC5B-AS1*1.60588 + TIPARP-AS1*1.343192 + RUSC1-AS1*1.585861 - LINC02315*2.66553 (**Table S7**). According to this cutoff value of risk score, the OS patients were classified into low- and high-risk groups. The risk score distribution and survival status plot demonstrated that the death cases were mainly distributed in the high-risk group (**Figure 2B**). The K-M survival analysis indicated that the OS patients in the low-risk group showed a significantly better overall survival than those in the high-risk group (**Figure 2C**). The areas under the curve (AUC) of the ROC curve remained above 0.75 at 1, 3 and 5 years, which means the novel CRLs signature had predictive performance in predicting survival risk (**Figure 2D**). Notably, we further performed a validation analysis in the testing cohort and the entire cohort to determine the predictive value of the novel CRLs signature. As expected, the validation results on the test cohort and the entire cohort are similar to those of the training set (**Figures S4, S5**). Together, these findings implied that the novel CRLs signature had a great prognostic prediction value for OS.

### The independent prognostic value of signature

**Figure 3:**
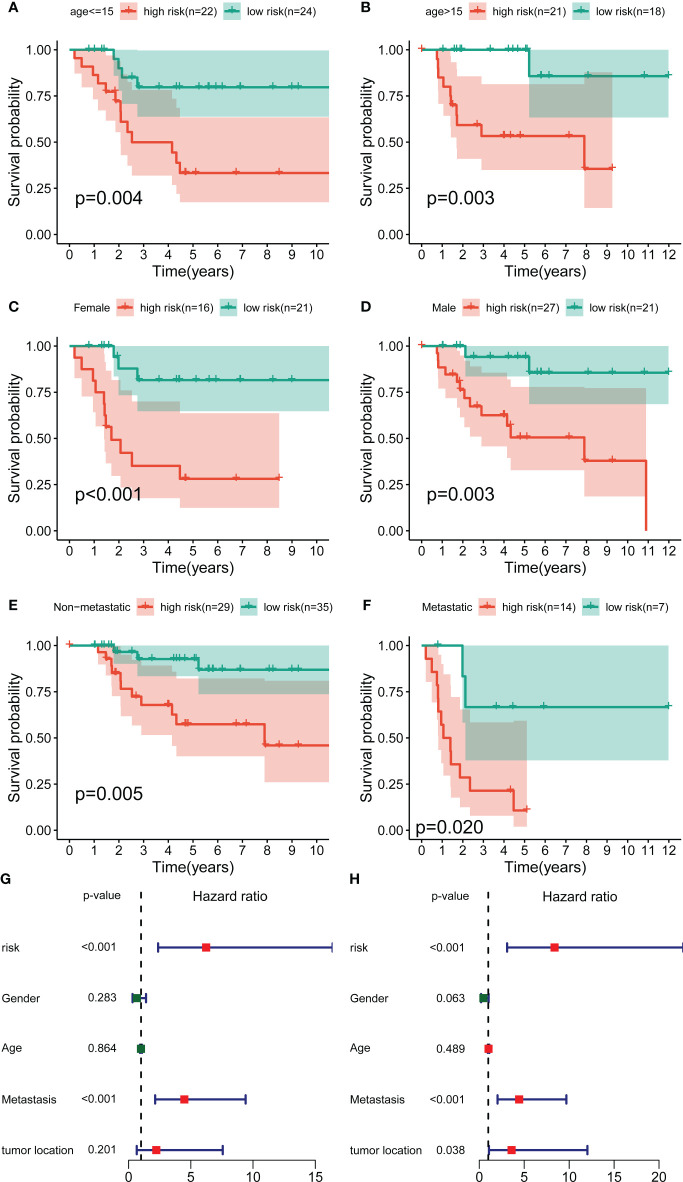
Independent prognostic value of the novel CRL signature. **(A–F)** K–M survival curves of overall survival stratified by age, gender, and metastasis status between low- and high-risk groups. **(G, H)** Univariate and multivariate Cox regression analyses demonstrated the novel CRL signature as an independent prognostic factor for OS.

To further exclude the impacts of other clinical characteristics on the prognostic values of the novel signature, we performed a subgroup survival analysis according to the baseline characteristics. As shown in **Figures 3A–F**, the OS cohort in the high-risk group had a worse prognosis than that in the low-risk group, regardless of the clinical subgroup. Further, the univariate Cox analysis demonstrated that the novel CRLs signature was associated with the prognosis of OS patients (HR: 6.233, 95%CI: 2.371-16.388) (**Figure 3G**). Besides, the multivariate Cox analysis further revealed that the signature was an independent factor for the prognosis of patients with OS (HR: 8.386, 95%CI: 3.098-22.701) (**Figure 3H**). Collectively, these results indicated that this novel CRLs lncRNA signature could reliably serve as an independent prognostic factor for OS.

### The association between signature genes with cuproptosis in OS

**Figure 4:**
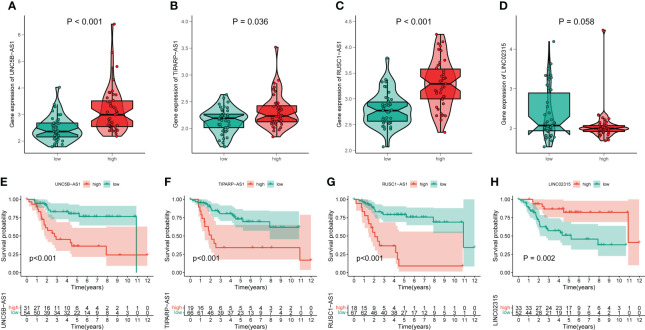
The association of these signature lncRNAs with OS. **(A–D)** The expression level of each signature lncRNAs in the low- and high-risk groups. Green and red represent the low-risk group and high-risk group, respectively. **(E–H)** The K-M survival curves for these four signature lncRNAs in OS.

By performing correlation analysis, we have seen that the four signature CRLs were closely related to cuproptosis-related genes (**Figure S6**). Also, there was a significant positive association between the four risk CRLs (**Figure S6**). Subsequently, to further explore the relationship between these four CRLs and the novel signature, we analyzed the expression levels of these four CRLs between different risk groups. We observed an upregulated expression level of UNC5B-AS1, TIPARP-AS1, and RUSC1-AS1 in the high-risk group compared to the low-risk group (**Figures 4A–C**). On the contrary, LINC02315 was downregulated in the high-risk group, albeit not statistically significantly (**Figure 4D**). Meanwhile, to assess the prognostic effects of each signature CRLs on OS, we performed KM survival analysis to investigate the relevance of these four CRLs to the prognosis of OS. We found that all four signature CRLs had pronounced prognostic effects in OS (**Figures 4E–H**). Among them, UNC5B-AS1, TIPARP-AS1, and RUSC1-AS1 were risk factors for OS, while LINC02315 was a protective gene for OS. In sum, it is confirmed that there was an independent relevance between each signature CRLs and the cuproptosis and prognosis of OS.

### Differentially expressed and functional enrichment analysis between distinct risk groups

**Figure 5:**
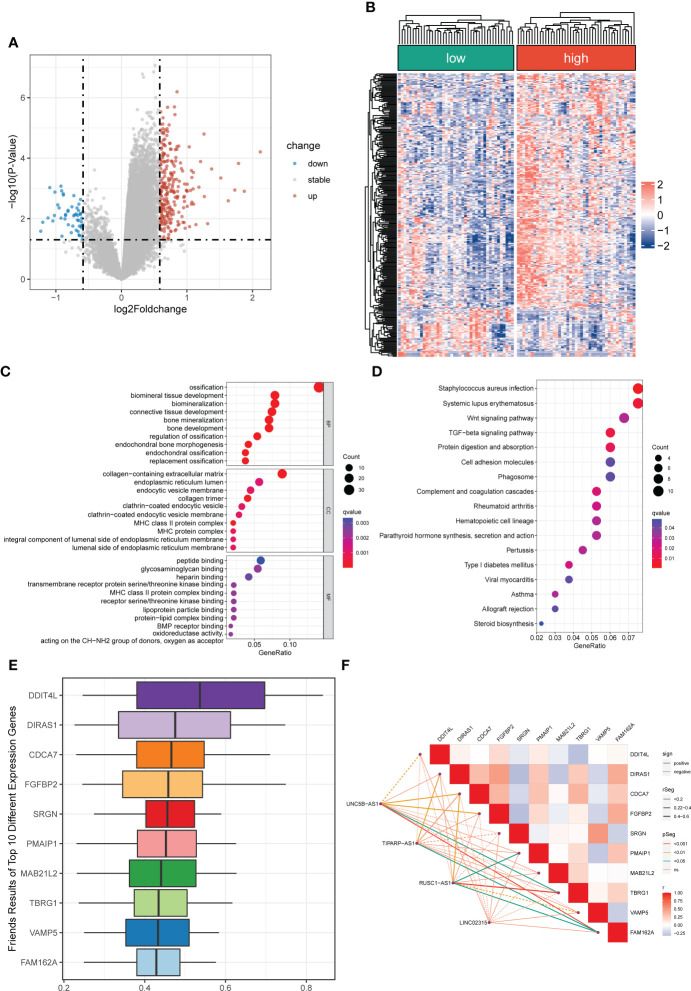
Differentially expressed and functional enrichment analysis between distinct risk groups. **(A, B)** The volcano plot and heatmap of differentially expressed genes between the low- and high-risk groups. **(C, D)** The GO analysis and KEGG enrichment pathway analysis between different risk groups. **(E)** The Friends analysis of GO-related genes. **(F)** The association between these ten hub genes and four signature lncRNAs.

A total of 296 differential expressed genes between high-risk and low-risk groups were identified by differential analysis (**Table S8**). The results demonstrated a marked difference in mRNA expression profiles between high- and low-risk groups (**Figure S7**). Also, we observed that most differential genes were upregulated in the high-risk group from the cluster heatmap and volcano plot (**Figures 5A, B**). Meanwhile, we performed a PPI network analysis of differentially expressed genes using the STRING database for subsequent hub genes identification (**Figure S7**). In addition, the GO and KEGG analyses were utilized to explore potential differences in biological functions and signaling pathways between different risk groups classified by the novel CRLs signature. The GO analysis showed that the CRLs signature was associated with bone metabolism-related functions (**Figure 5C**). The KEGG results indicated that the OS patients in the high-risk group were significantly enriched in some cancer-related pathways, such as the Wnt signaling pathway, TGF-beta signaling pathway, and Cell adhesion molecules (**Figure 5D**). Hence, these results implied a significant molecular functional difference between the two distinct risk groups and the novel CRLs signature was mainly associated with the tumorigenesis pathway in OS.

### Identification of cuproptosis-related hub genes

To further identified potential hub genes associated with cuproptosis, we screened the top ten hub genes using Friends analysis. These ten hub genes may play potential roles in the molecular functional processes relevant to cuproptosis (**Figure 5E**). Meanwhile, there was a pronounced co-expression relationship between these ten hub genes and the signature CRLs (**Figure 5F**). Additionally, to further investigate the survival impact of these hub genes in OS, we used K-M survival analysis to detect the correlation between the expression of each gene and the prognosis of OS. Also, the AUC of the ROC curve was calculated to evaluate the predictive performance of each hub gene. From the survival analysis and ROC curve, it can be seen that the overexpression of DIRAS1, CDCA7, FGFBP2, PMAIP1, TBRG1, and FAM162A predicted poor prognosis and had good predictive performance (**Figures S8, S9**). However, the predictive role of DDIT4L, SRGN, VAMP5, and MAB21L2 for OS needs to be further confirmed in the future (**Figures S8, S9**).

### The immune features between distinct risk groups

**Figure 6:**
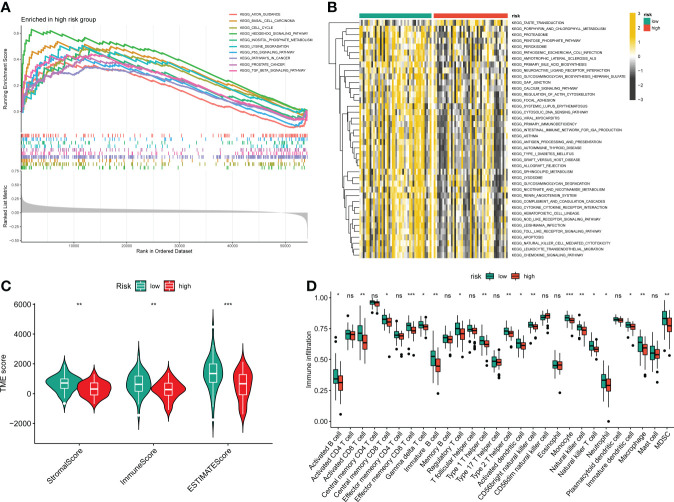
The relationship between the novel CRLs signature and tumor immune microenvironment in OS. **(A)** The GSEA between the two distinct risk groups. **(B)** The heatmaps of GSVA displayed signaling pathways between the high-risk and low-risk groups. **(C)** The TME score between distinct groups. **(D)** Box plot indicating the relative abundance of immune cells based on ssGSEA in the distinct risk groups. *p < 0.05, **p < 0.01, ***p < 0.001.

To further determine the molecular functional differences between high- and low-risk groups, we implemented GSEA and GSVA. The GSEA result showed that the high-risk groups also enriched several tumor-related pathways, which further confirmed that the novel CRLs signature was relevant to the development of OS (**Figure 6A**). Also, the GSVA demonstrated the OS patients with lower CRL risk scores were significantly enriched in immune-activated pathways, such as antigen process and presentation, cytokine-cytokine receptor interaction, Nod-like receptor interaction, and TOLL-like receptor signaling pathway, implying there was a significant difference in the tumor immune microenvironment between the two distinct groups (**Figure 6B**). Therefore, we investigated the role of the CRLs signature in the immune microenvironment of OS. Initially, the ESTIMATE analysis displayed that the low-risk group had a higher TME score (stromal score, immune score, and estimate score) than the high-risk group (**Figure 6C**). For the TME score, higher stromal scores or immune scores represented larger amounts of immune or stromal cellular components in the TME, while estimate scores indicated the sum of stromal or immune scores. Similarly, the ssGSEA revealed significant differences in the infiltration of most immune cells between the two risk groups (**Figure 6D**). The activated B cell, activated CD8 T cell, activated dendritic cell, CD56bright natural killer cell, central memory CD8 T cell, effector memory CD8 T cell, gamma delta T cell, immature B cell, immature dendritic cell, macrophage, MDSC, monocyte, natural killer cell, natural killer T cell, neutrophil, regulatory T cell, type 1 T helper cell, and type 2 T helper cell showed a more significant infiltration in the CRLs low-risk groups (**Figure S10**). In addition, the four signature CRLs and most cuproptosis-related hub genes were negatively relevant to the infiltration of immune cells (**Figure S11**). Ultimately, the heatmap revealed no significant differences between the two distinct risk groups in terms of clinical characteristics (**Figure S11**). Altogether, these findings suggested that the activated immune status may account for the better prognosis of OS in the low-risk group.

### Immunotherapy and chemotherapy drugs response

**Figure 7:**
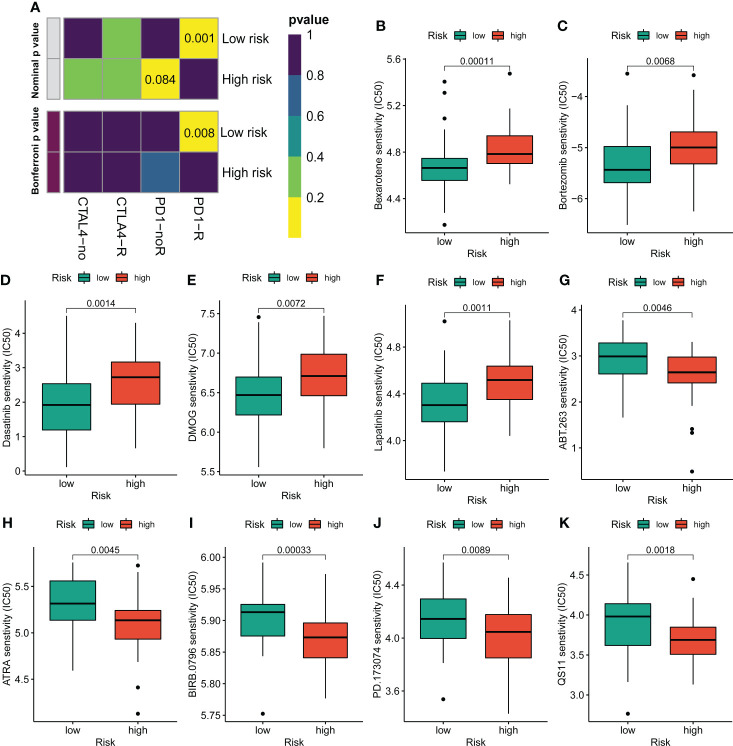
Assessment of immunotherapy and chemotherapy response efficacy in patients from different risk groups. **(A)** Sensitivity prediction of distinct groups to the two immune checkpoint inhibitors in the OS cohort. No response, noR; response, R. **(B–K)** The sensitivity to chemotherapeutic drugs was represented by the half-maximal inhibitory concentration (IC50) of chemotherapeutic drugs.

Given the clinical role of immunotherapy and chemotherapy in OS, we then explored the relationship between the CRL risk score and immunotherapy response and drug sensitivity. Briefly, the submap demonstrated that the OS patients with low CRL risk scores were more likely to respond to anti-PD1 therapy, which may provide new insight into the immunotherapy for OS (**Figure 7A**). Next, we compared the difference in sensitivity to anti-tumor agents between the two distinct risk groups. As shown in **Figures 7B–F**, the patients in the low-risk group had lower IC50 values for Bexarotene, Bortezomib, Dasatinib, DMOG, and Lapatinib, which means these low-risk patients have a better response to these drugs. In contrast, the patients with high-risk scores were more likely to respond to ABT.263, ATRA, BIRB.0796, PD.173074, and QS11(**Figures 7G–K**). Overview, these findings demonstrate that the novel CRLs signature may help to predict the efficacy of immunotherapy and chemotherapy.

### Construction of the nomogram

**Figure 8:**
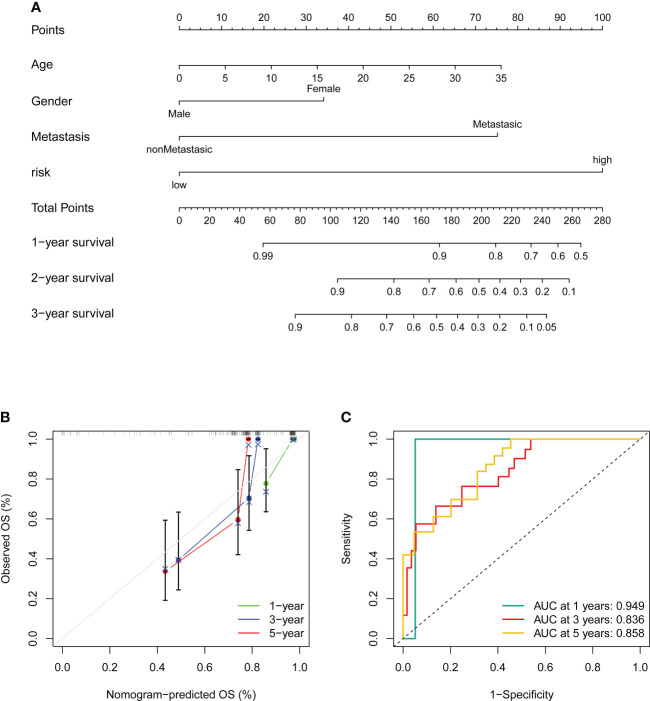
Construction and validation of a nomogram. **(A)** Nomogram for predicting the 1-, 3-, and 5- overall survival of OS patients. **(B)** Calibration curves of the nomogram for predicting the 1-, 3-, and 5- overall survival of OS patients. The dashed diagonal line in grey colour represents the ideal nomogram. **(C)** The ROC curves of the nomogram estimate the prognostic value of the nomogram.

To better utilize the novel CRLs signature for clinical application, we constructed a predictive cuproptosis-related prognostic nomogram based on the clinical characteristics, including age, gender, metastasis, and the CRL risk score. As presented in **Figure 8A**, the nomogram could predict the 1-, 3-, and 5-year probably overall survival rate. Notably, the prognosis predictive performance for OS is shown by the calibration curves. The calibration curve for the predictive probability showed that the nomogram-predicted overall survival was in accordant agreement with the actually observed overall survival of OS (**Figure 8B**). Additionally, the 1-, 3-, and 5-year AUC values of the nomogram were 0.949, 0.836, and 0.858, respectively (**Figure 8C**). Hence, these results imply that the CRLs prognostic signature was stable and accurate, which may be used in the clinical management of OS patients.

### Validation of the expression of signature lncRNAs

**Figure 9:**
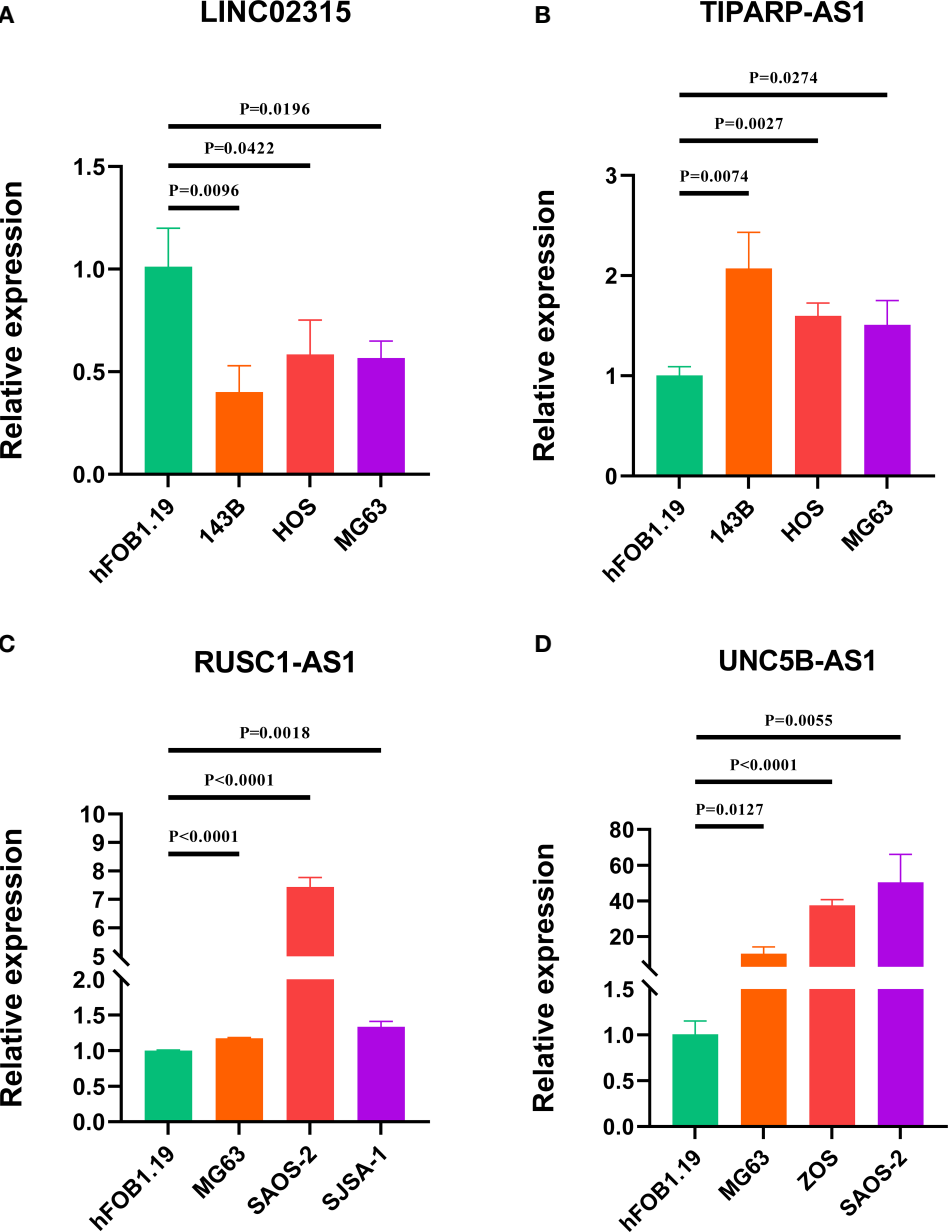
Verification of the expression of signature CRLs in OS cell lines by using RT-qPCR. **(A)** LINC02315. **(B)** TIPARP-AS1. **(C)** UNC5B-AS1. **(D)** RUSC1-AS1.

To further evaluate the expression level of these four signature CRLs, we utilized their expression in the cell lines using RT-qPCR. As presented in **Figures 9A, B**, compared with those in the normal cell line (hFOB1.19), LINC02315 was downregulated in the OS cell line (including 143B, HOS, and MG63), while TIPARP-AS1 exhibited the opposite trend in 143B, HOS, and MG63 cell line. In addition, the UNC5B-AS1 and RUSC1-AS1 had a higher expression level in the OS cell line than in the normal cell line (**Figures 9C, D**). In sum, these results further validated the previous accuracy of our previous bioinformatics analysis.

## Discussion

In the present study, we identified 120 differentially expressed CRLs in OS *via* Pearson correlation and differential expression analysis. Then, we obtained 31 CRLs associated with the prognosis using univariate regression analysis and constructed a CRL prognostic signature consisting of four prognostic CRLs by LASSO and multivariate regression analysis. Next, the novel CRL signature was evaluated utilizing survival analysis, ROC curve, internal validation, independent prognostic analysis, and *in vitro* experiments. As far as we are aware, our research first analyzed the CRLs in OS systematically and comprehensively, revealing that the novel CRL signature could be used as a promising prognostic indicator for OS. Subsequently, we found that DIRAS1, CDCA7, FGFBP2, PMAIP1, TBRG1, FAM162A, DDIT4L, SRGN, VAMP5, and MAB21L2 are hub genes in the altered process between the two distinct risk groups. Interestingly, these genes are known to play a crucial role in tumorigenesis and development. In particular, Huan Liu et al. reported that DIRAS1 regulated by METTL3 and METTL14 could control the malignant progress of OS *via* regulating the ERK pathway (25). While the effects of CDCA7, FGFBP2, and FAM162A on OS remain unclear, their role in tumors has been documented during the last few years (26–28). For instance, CDCA7 regulate the expression of CCNA2 to facilitate the tumor progression of Esophageal Squamous Cell Carcinoma (28). Given the above analyses and previous investigations, these hub genes may be a potential molecular target for the modulation of cuproptosis in OS.

Next, the KEGG and GSEA demonstrated that the OS patients in the CRL high-risk group were primarily enriched in tumor-associated biological processes, such as the Wnt signaling pathway, TGF-beta signaling pathway, and Cell adhesion molecules. These pathways have been documented to be associated with OS aggressive phenotypes. For example, NGX6 could inhibit the viability, invasion, and migration, while promoting the apoptosis of OS cell lines by suppressing the Wnt/β-catenin signaling pathway (29). This result suggests that the novel CRL signature in OS may be involved in these tumor-related signaling pathways. To further evaluate the tumor immune microenvironment in OS, we calculated the tumor microenvironment score and the degree of infiltration of different types of immune cells. Previous studies revealed that cancer patients with higher immune and stromal scores have an improved prognosis (30, 31). Consistently, our results exhibited that the low-risk OS patients had higher immune, stroma, and ESTIMATE scores than those in the high-risk group. Similarly, the ssGSEA results indicated that the infiltration rate of most immune cells in the high-risk group was lower than in the low-risk group. Some of them were reported to be tumor antagonistic immune cells (32, 33). In a previous study, the infiltration of activated CD8 T cells was proven to correlate to improved prognosis and survival of primary ovarian cancer (34). Furthermore, we also found that the four CRLs were positively relevant to the abundance of immune cells, implying the novel CRL signature could be used to assess the tumor immune microenvironment in OS. Therefore, it is reasonable to believe that a better tumor immune microenvironment may partly account for a better prognosis of OS patients in the low-risk group. Blocking the immune checkpoint therapy (such as PD-1 and CTLA-4) is a promising approach for various cancer (35). In the present study, we surprisingly found that the OS patients with lower risk scores are promising in response to anti-PD-1, suggesting that the novel CRL signature might be an underlying index for evaluating immunotherapy response in OS. In addition, we assessed the susceptibility to chemotherapeutics between the two distinct groups. We observed that the OS patients with different risk scores are different in sensitivity to chemotherapeutic drugs, which is beneficial in providing appropriate treatment options for OS. Importantly, we verified these four signature CRLs expressions in the OS cell. The expression trend was consistent with the previous bioinformatic analysis, which further proves the validity of the novel CRL signature. It is worth mentioning that some of these signature CRLs had been confirmed to play different functional roles in tumor progression. For instance, UNC5B-AS1 promotes the malignant progression of hepatocellular carcinoma, ovarian cancer, prostate cancer, lung cancer, and thyroid cancer (36–40). Notably, the tumor promotion role of RUSC1-AS1 has been well-characterized in OS (41, 42). Rui Jiang et al. reported that RUSC1-AS1 facilitate the malignancy of OS *via* the miR-101-3p-regulating Notch1 signaling pathway (41). As a candidate protective factor for lung squamous cell carcinoma, LINC02315 was diminished in lung squamous cell carcinoma tissue and had great predictive efficiency (43). Still, studies regarding the significance of TIPARP-AS1 in tumor development are not fully known. In our study, we observed that TIPARP-AS1 was elevated in OS cells and correlated with a poor prognosis of OS, but the specific roles of TIPARP-AS1 in OS need further exploration. Based on the above results and previous research, it is reasonable to believe the reliability of the novel CRLs signature.

Despite these promising findings, it is worth noting that some limitations do exist. First, the prognostic value of the novel CRLs signature needs to be further validated in prospective studies and clinical cohorts. In addition, the specific mechanisms of these signature CRLs in OS should be further confirmed using *in vivo* and *in vitro* experimental validation and further explore the relationship between the novel signature and tumor immunity microenvironment. We will incorporate these efforts into future studies.

## Conclusion

In conclusion, we establish a novel signature composed of four CRLs that could robustly predict the prognosis of OS. In addition, the relationships between the CRL signature and tumor immune microenvironment, immunotherapy and chemotherapy response were preliminarily ascertained. It is reasonable to believe that our study may provide valuable insights into clinical decision-making and personalized therapeutic regimens basis for future research.

## Data availability statement

The original contributions presented in the study are included in the article/**Supplementary Material**. Further inquiries can be directed to the corresponding authors.

## Author contributions

SSH and CT contributed to the conception and made finalapproval of the version, BFL performed study concept and designand wrote the manuscript. ZYL performed the experiment. ZYL,CYF, CBL, HXZ, and ZHL helped with data analysis. All authorscontributed to the article and approved the submitted version.

## Funding

This work was supported by the National Natural Foundation of China (81902745), Hunan Provincial Natural Science Foundation of China (2022JJ30843), the Science and Technology Development Fund Guided by Central Government (2021Szvup169), and Hunan Provincial Administration of Traditional Chinese Medicine Project (No. D2022117).

## Conflict of interest

The authors declare that the research was conducted in the absence of any commercial or financial relationships that could be construed as a potential conflict of interest.

## Publisher’s note

All claims expressed in this article are solely those of the authors and do not necessarily represent those of their affiliated organizations, or those of the publisher, the editors and the reviewers. Any product that may be evaluated in this article, or claim that may be made by its manufacturer, is not guaranteed or endorsed by the publisher.

## Abbreviations

OS, osteosarcoma; CRL, cuproptosis-related lncRNA; LASSO, least absolute shrinkage and selection operator; K-M, Kaplan–Meier; DLAT, dihydrolipoamide S-acetyltransferase; TCA, tricarboxylic acid; lncRNA, long non-coding RNA; GSEA, gene set enrichment analysis; GSVA, gene set variation analysis; GEO, Gene Expression Omnibus; TARGET, Therapeutically Applicable Research To Generate Effective Treatments; PCA, principal component analysis; ROC, receiver operating characteristic; GO, Gene Ontology; KEGG, Kyoto Encyclopedia of Genes and Genomes; BP, Biological Process; CC, Cellular Component; MF, Molecular Function; PPI, protein-protein interaction; ESTIMATE, Estimation of Stromal and Immune Cells in Malignant Tumors using the Expression Data; ssGSEA, single sample gene set enrichment analysis; Submap, subclass mapping; IC50, half maximal inhibitory concentration; ATCC, American Type Culture Collection; DMEM/F12, Dulbecco’s Modified Eagle Medium/Nutrient Mixture F12; MEM, minimum essential medium; DMEM, Dulbecco’s Modified Eagle’s medium; RT-qPCR, Real Time Quantitative Polymerase Chain Reaction; AUC, areas under the curve.
