# Supplementary material for: Identification of cuproptosis-related lncRNA prognostic signature for osteosarcoma
Source: Front Endocrinol (Lausanne). 2022 Oct 13;13:987942. doi: 10.3389/fendo.2022.987942 (PMC9606239; doi:10.3389/fendo.2022.987942)
Supplement: Supplementary file 1 [file DataSheet_1.docx]

**Supplementary Figures**

**
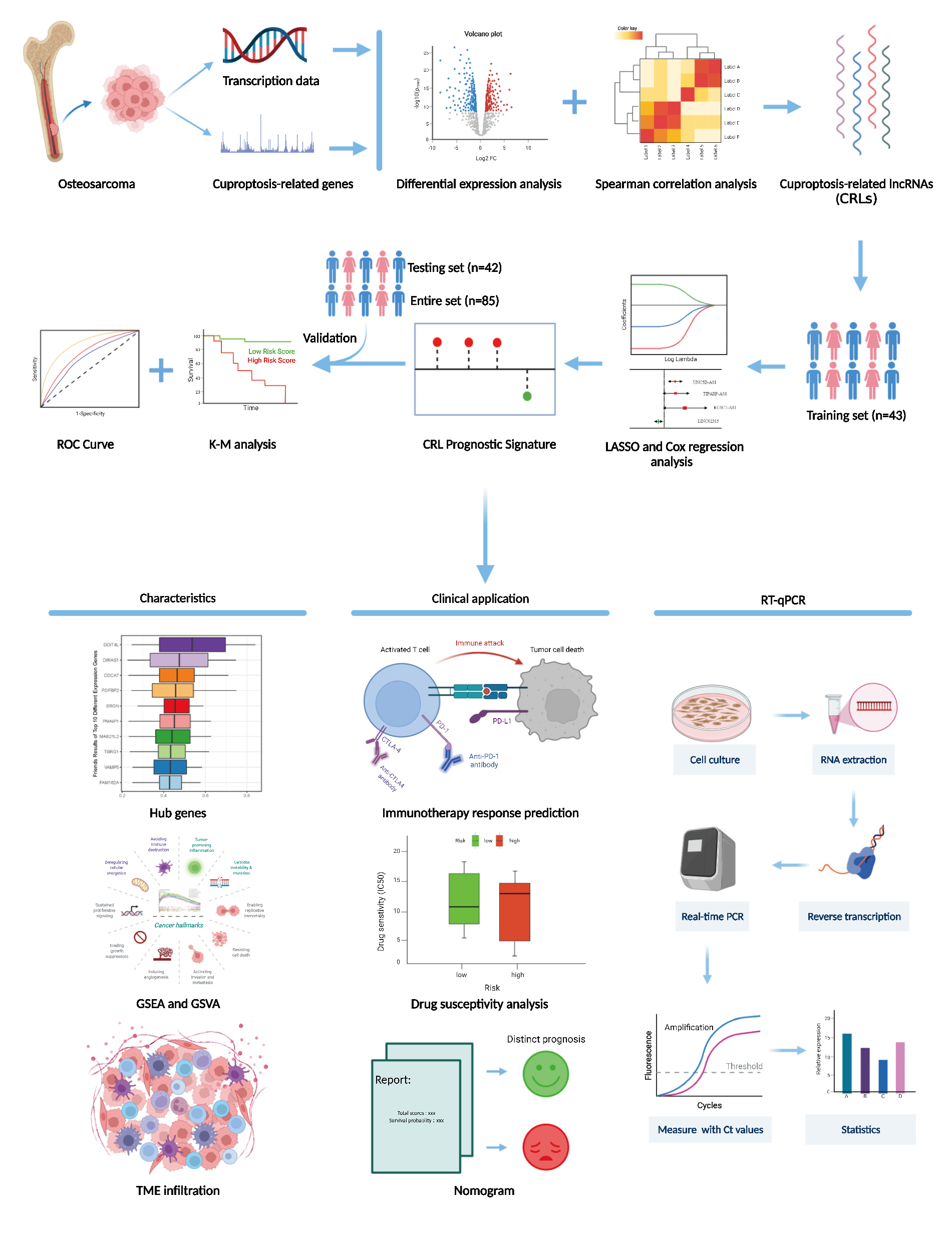
**

**Figure S1.** The flowchart of the present study.


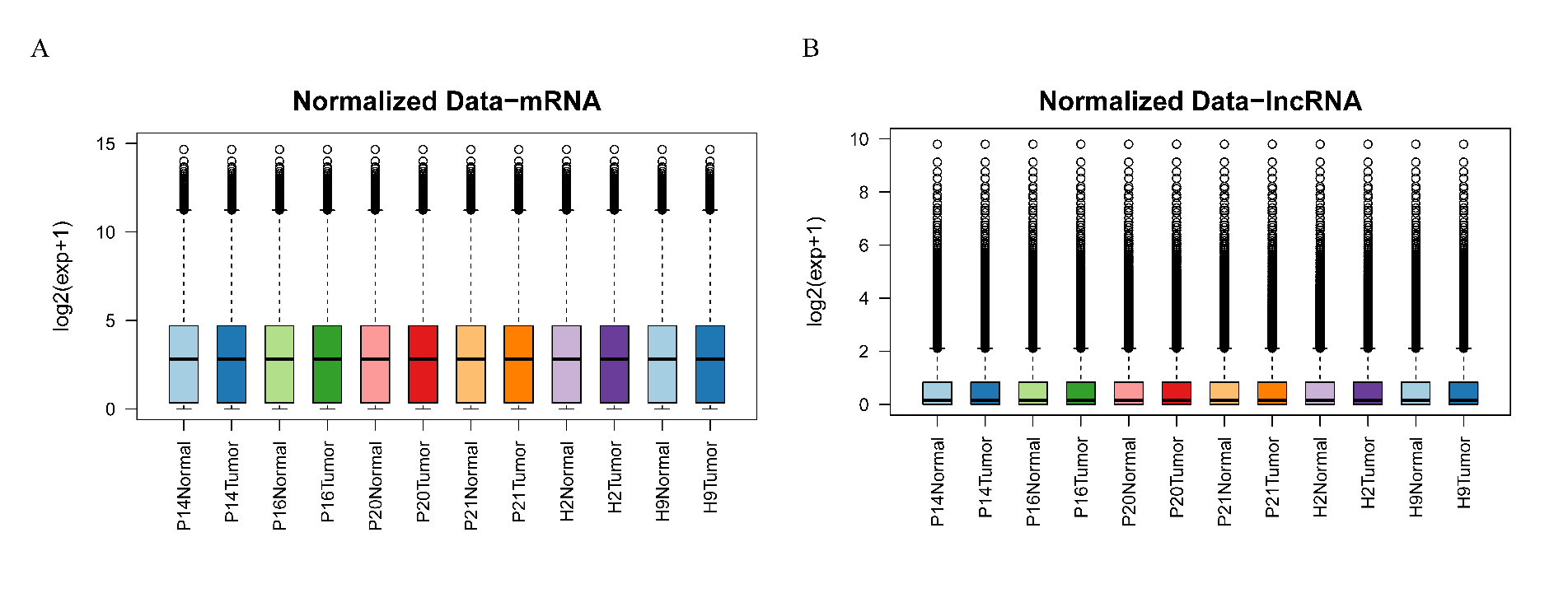


**Figure S2.** Boxplots of mRNA and lncRNA expression data after batch effect correction.


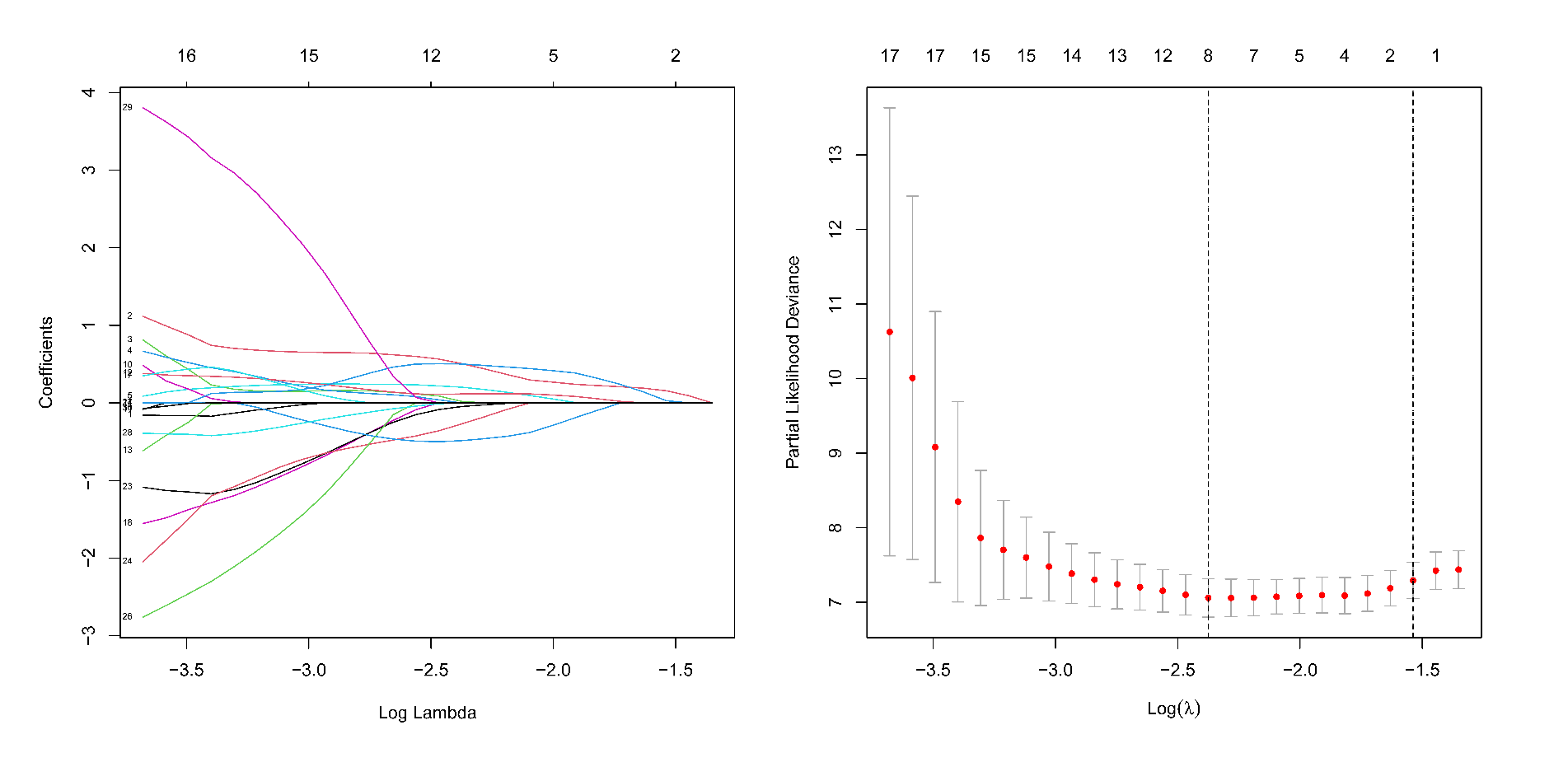


**Figure S3.** Cvfit and lambda curves showing the least absolute shrinkage and selection operator (LASSO) regression was performed with the minimum criteria.


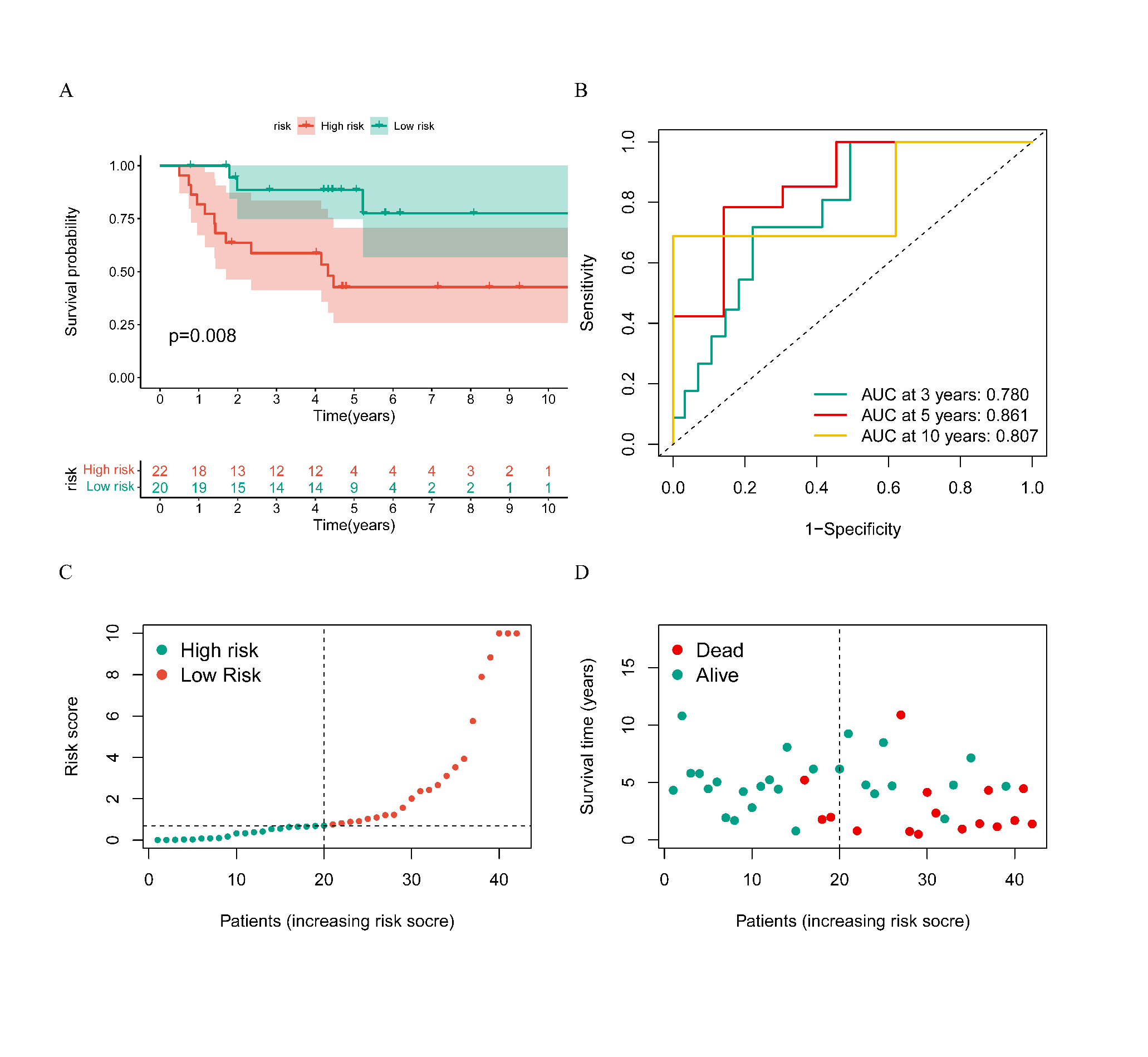


**Figure S4.** Validation of the novel CRLs signature in testing set. (A) The KM analysis of the overall survival between the two different risk groups in the testing groups. (B) ROC curves verified the prognostic performance of the novel signature in the testing groups. (C-D) The distribution of the risk scores and the distributions of overall survival status and risk score in the testing groups.


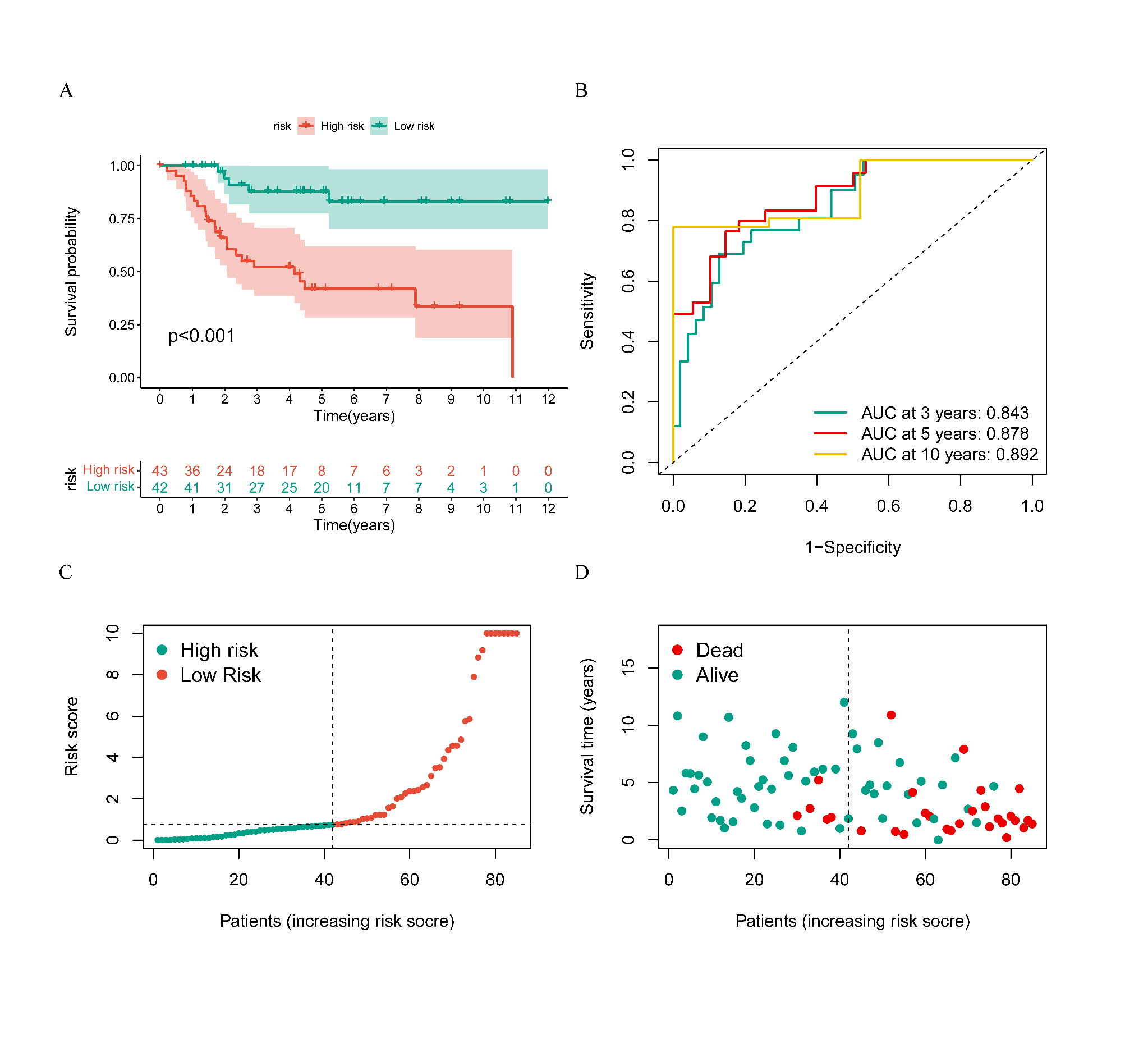


**Figure S5.** Validation of the novel CRLs signature in entire set. (A) The KM analysis of the overall survival between the two different risk groups in the entire groups. (B) ROC curves verified the prognostic performance of the novel signature in the entire groups. (C-D) The distribution of the risk scores and the distributions of overall survival status and risk score in the entire groups.


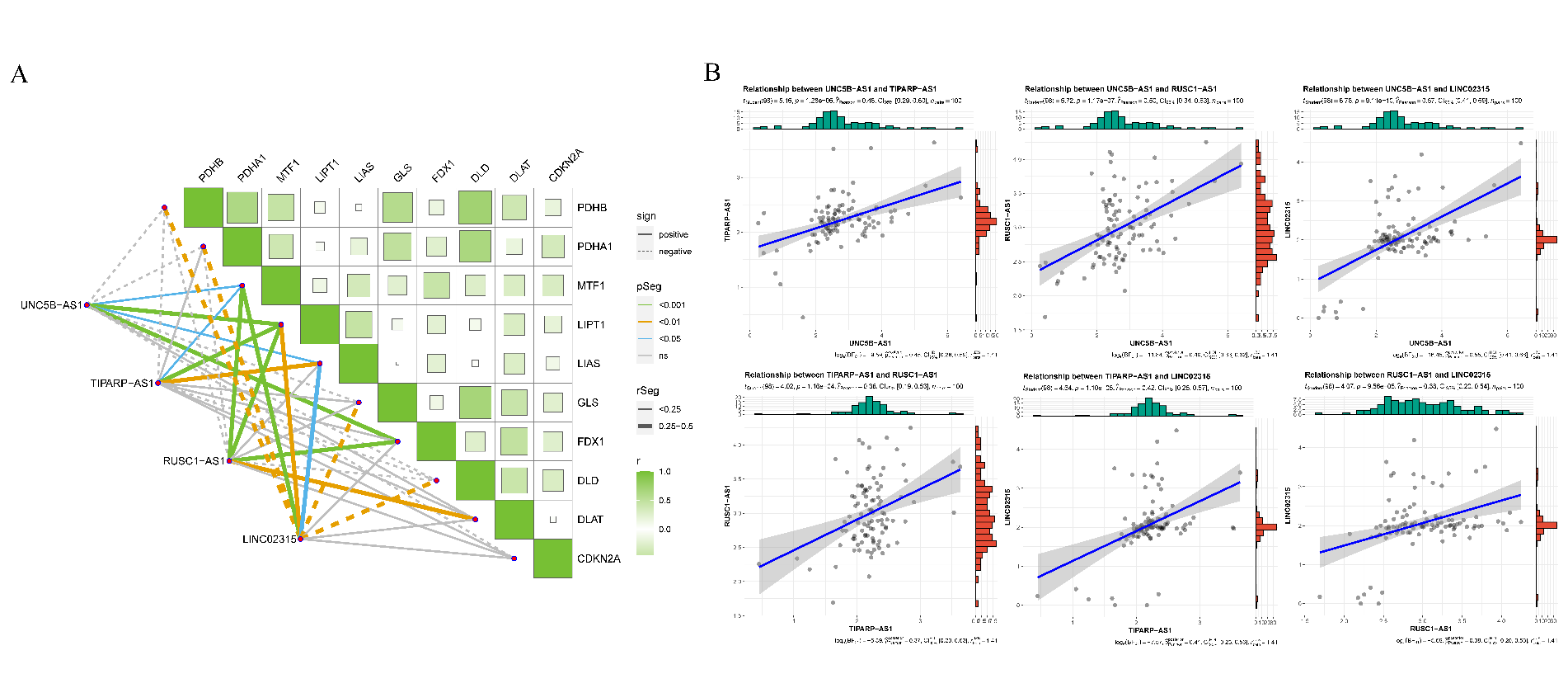


**Figure S6.** The association of these signature lncRNAs with cuproptosis. (A) The co-expression analysis of the signature lncRNAs with cuproptosis-related gene. (B) The co-expression analysis of these signature lncRNAs with each other.


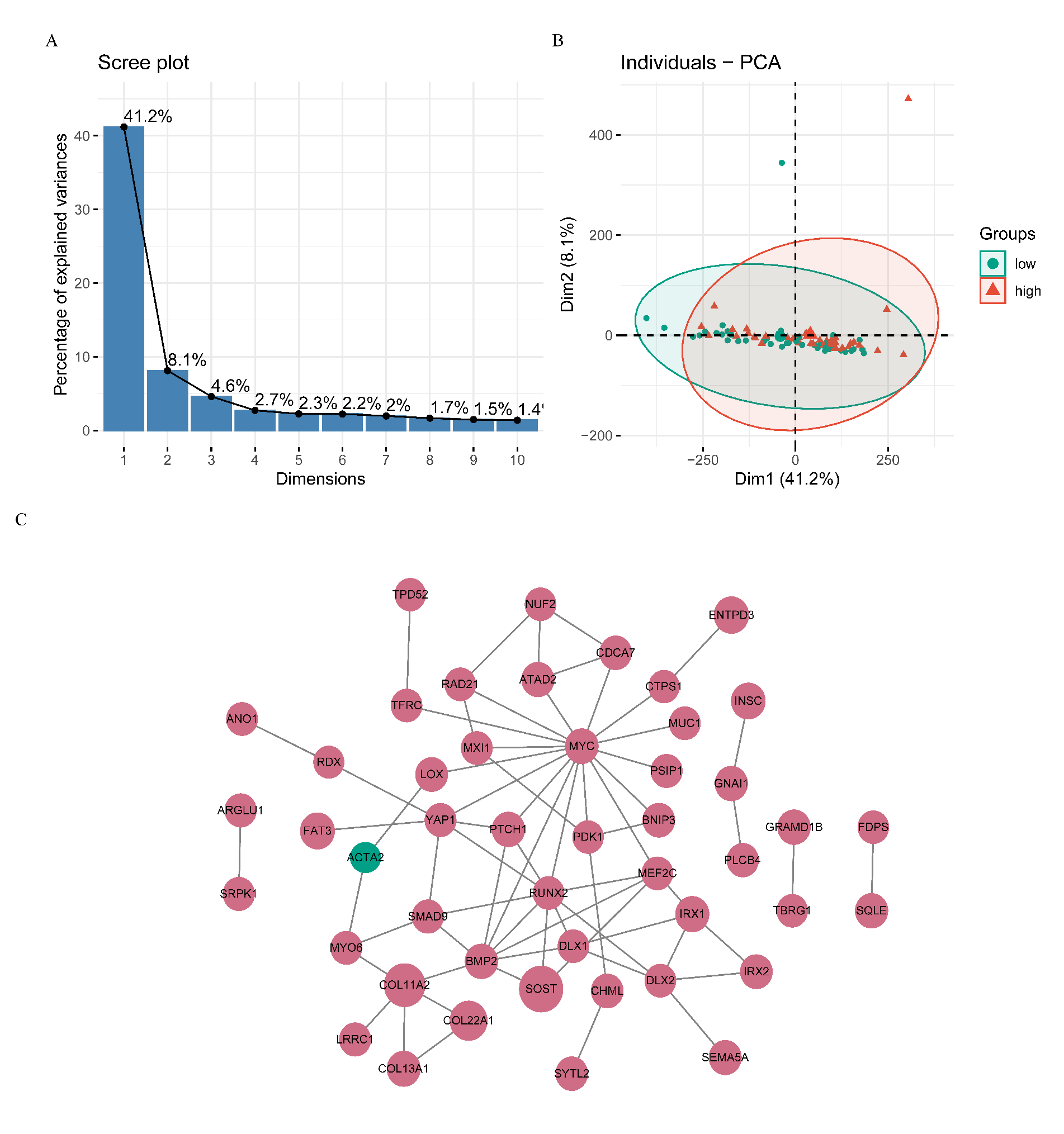


**Figure S7.** Differentially expressed gene between distinct risk groups. (A) The screen plot of PCA analysis is based on the differentially expressed gene between the low- and high-risk group. (B) PCA between high- and low-risk groups based on all genes. (C) PPI network analysis of differential mRNAs.


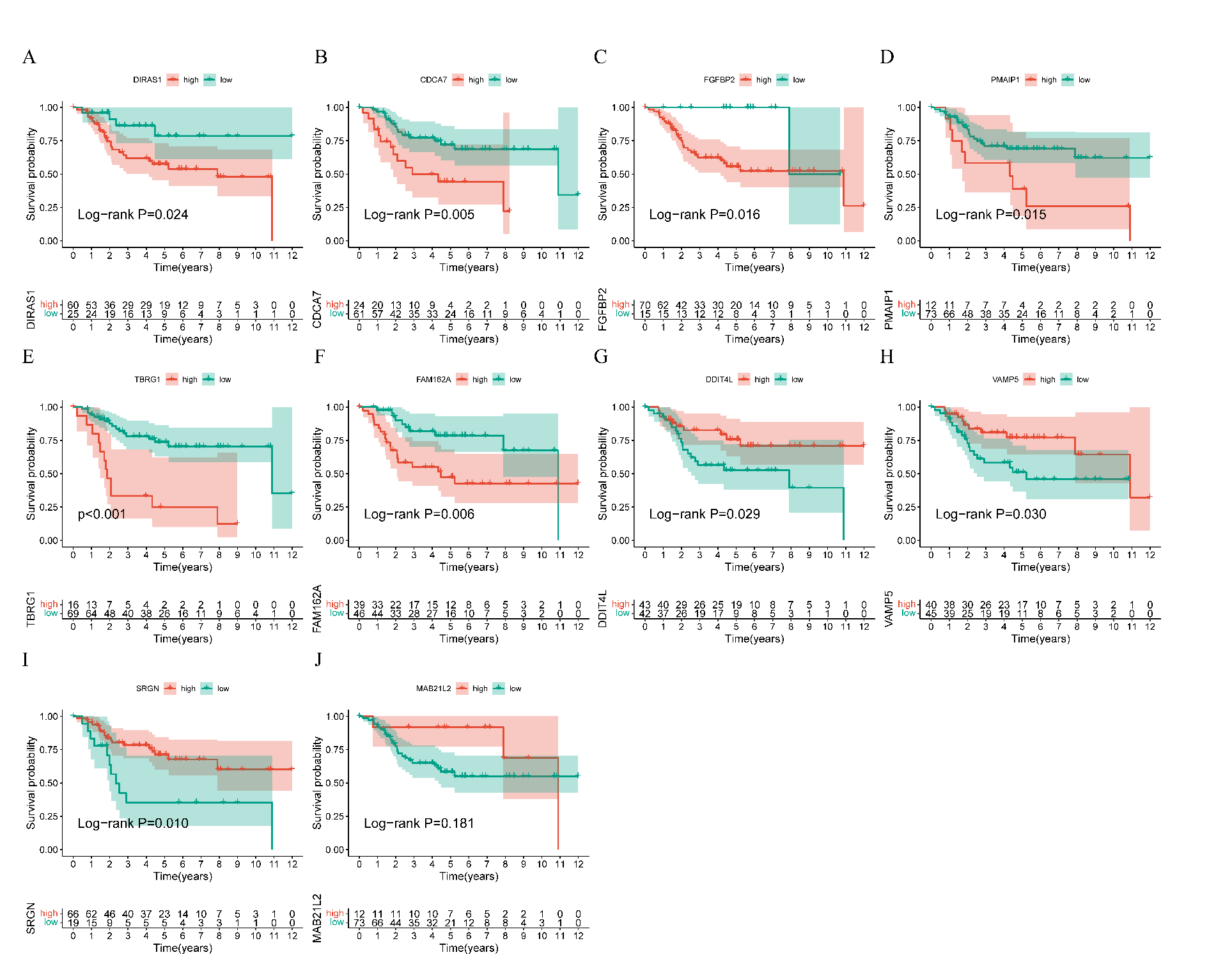


**Figure S8.** The K-M survival curve of these ten hub genes. (A) DIRAS1. (B) CDCA7. (C) FGFBP2. (D) PMAIP1. (E) TBRG1. (F) FAM162A. (G) DDIT4L. (H) VAMP5. (I) SRGN. (J)MAB21L2.


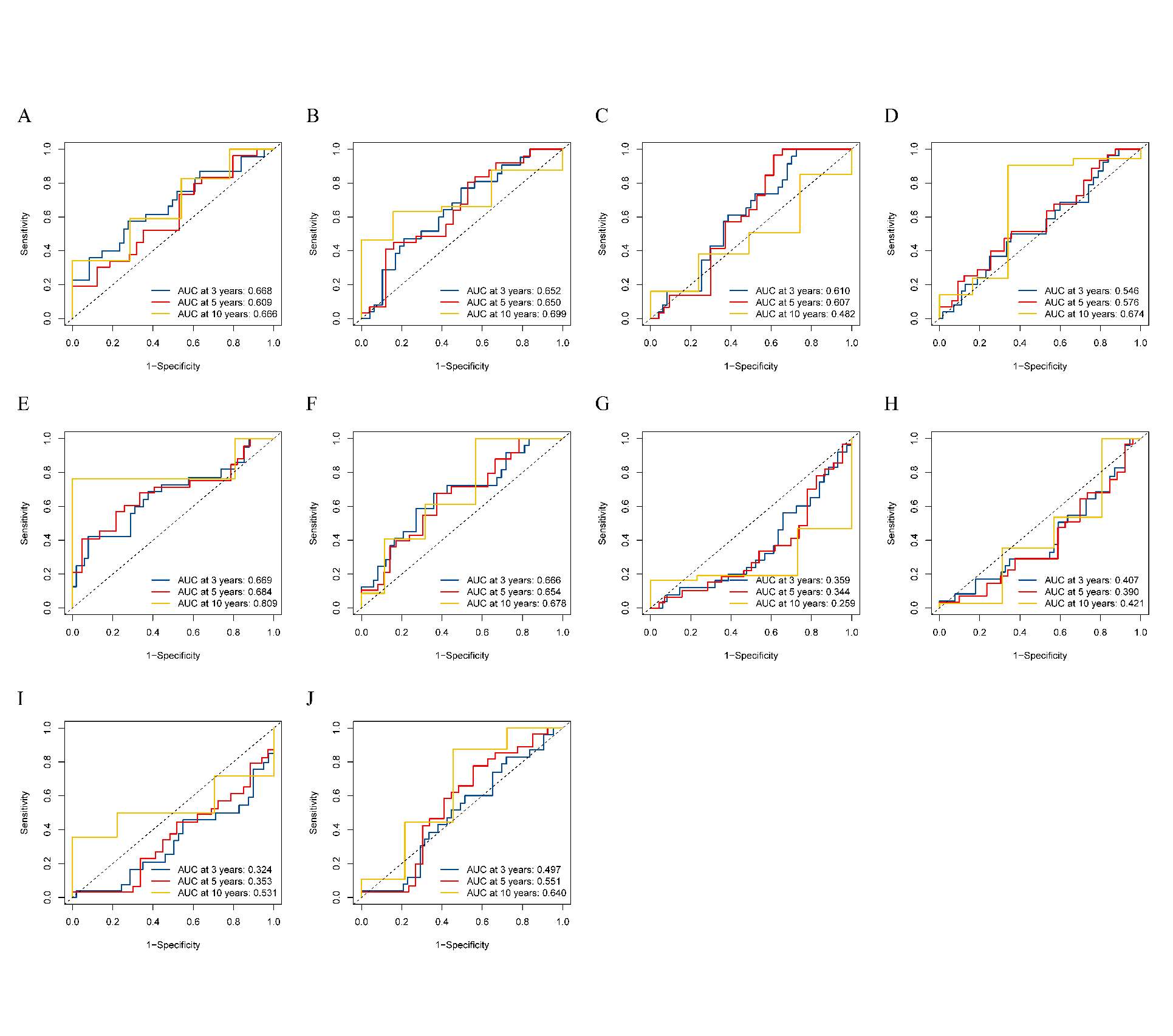


**Figure S9.** The ROC curve of these 10 hub genes. (A) DIRAS1. (B) CDCA7. (C) FGFBP2. (D) PMAIP1. (E) TBRG1. (F) FAM162A. (G) DDIT4L. (H) VAMP5. (I) SRGN. (J)MAB21L2.

**
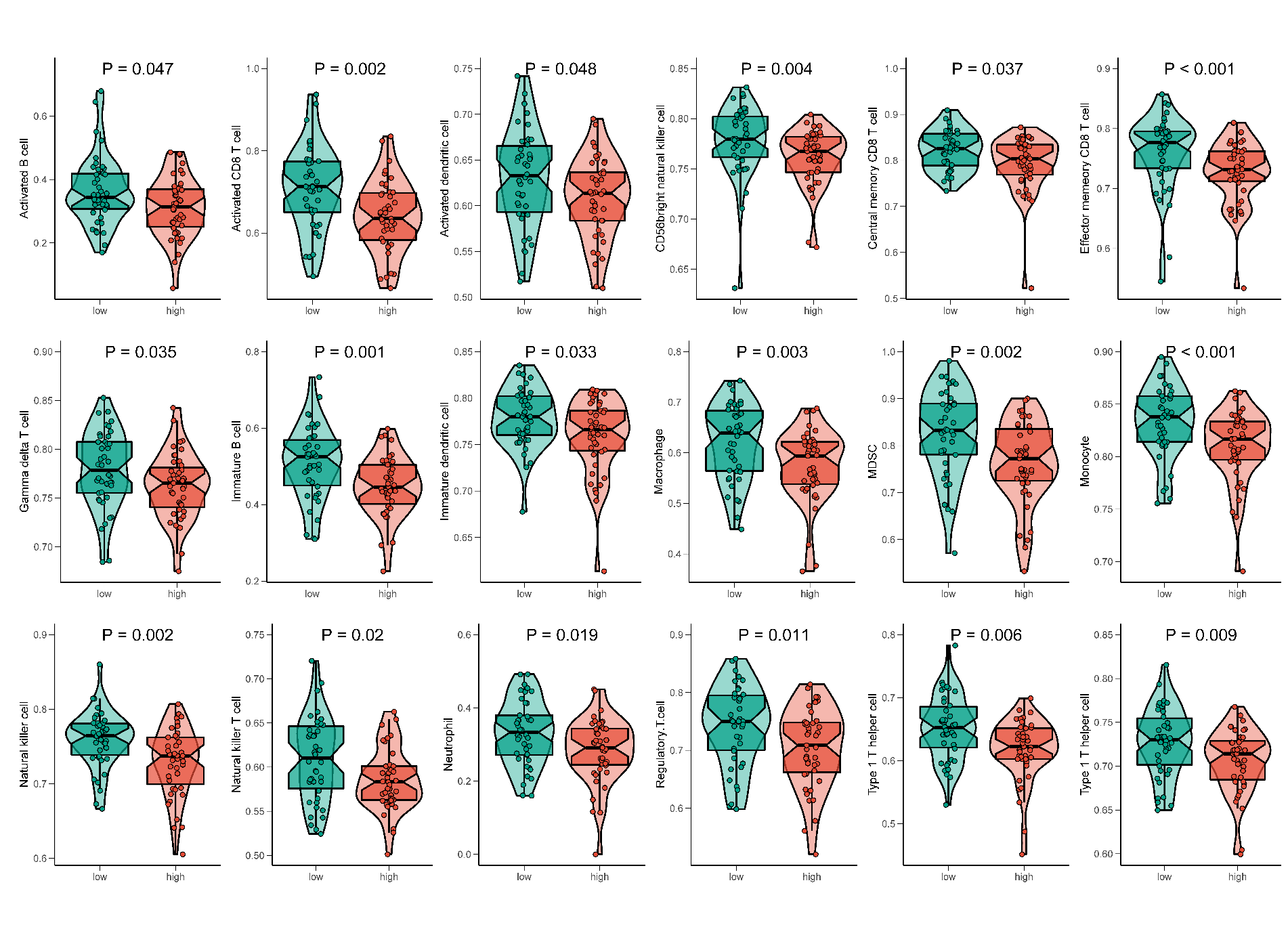
**

**Figure S10.** The difference in the proportion of activated B cell, activated CD8 T cell, activated dendritic cell, CD56bright natural killer cell, central memory CD8 T cell, effector memory CD8 T cell, gamma delta T cell, immature B cell, immature dendritic cell, macrophage, MDSC, monocyte, natural killer cell, natural killer T cell, neutrophil, regulatory T cell, type 1 T helper cell, and type 2 T helper cell between the different risk groups.


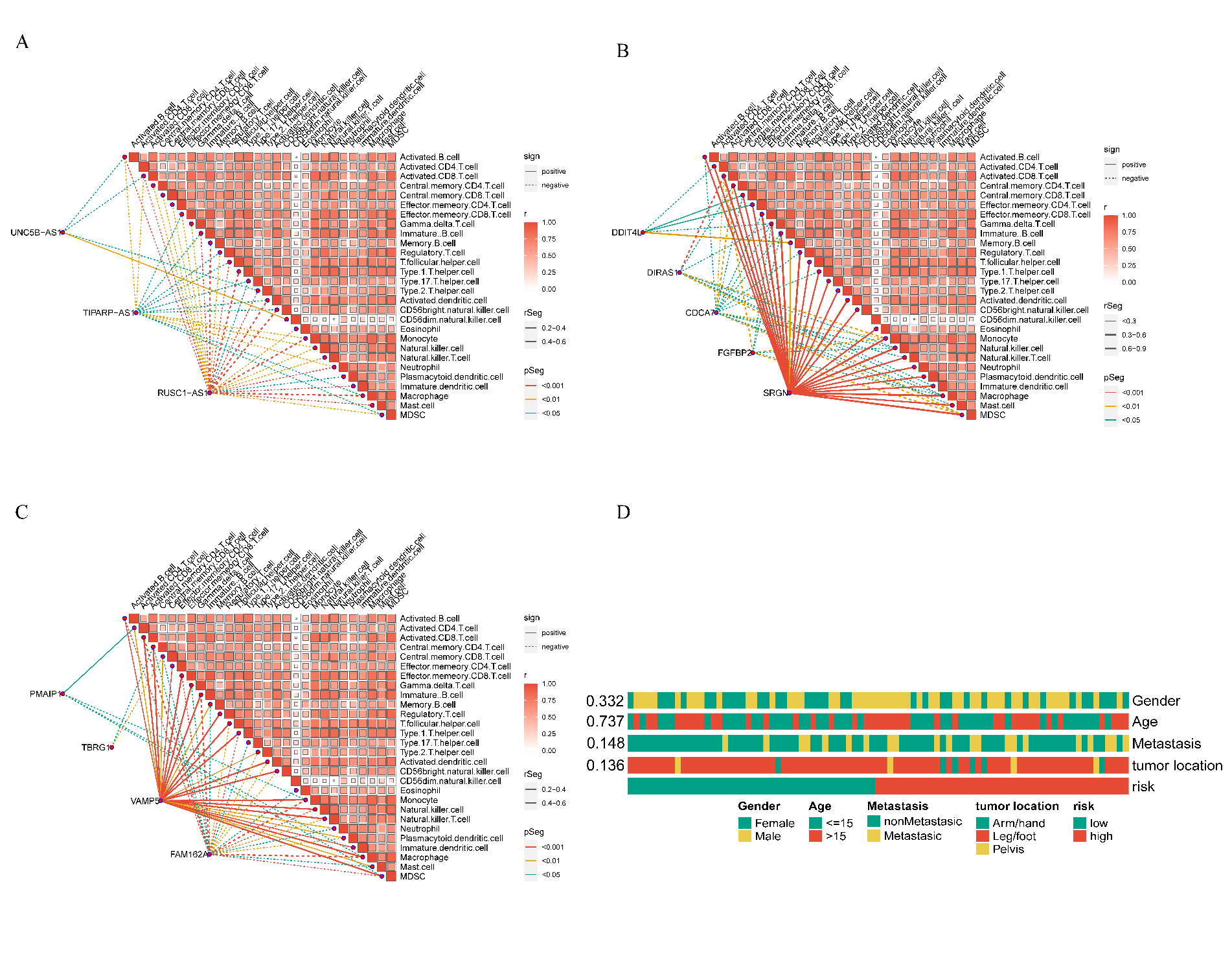


**Figure S11.** The relationship between the identified cuproptosis-related gene and tumor immune cell infiltration and clinical characteristics. (A-C) Correlation of CRLs and ten hub genes with immune infiltration cells in OS. (D) Association between the risk scores and clinical features.
